# Relationship between Anthropometric Parameters and Throwing Speed in Amateur Male Handball Players at Different Ages

**DOI:** 10.3390/ijerph17197022

**Published:** 2020-09-25

**Authors:** Jaime Tuquet, Juan Carlos Zapardiel, Jose M. Saavedra, Diego Jaén-Carrillo, Demetrio Lozano

**Affiliations:** 1 Health Sciences Faculty, Universidad San Jorge, Autov A23 km 299, 50830 Villanueva de Gállego, Zaragoza, Spain; jtuquet@usj.es (J.T.); djaen@usj.es (D.J.-C.); 2 Biomedical Science Department, Alcala University, 28054 Alcalá de Henares, Madrid, Spain; juancarloszapardiel@gmail.com; 3 Physical Activity, Physical Education, Sport and Health (PAPESH) Research Centre, Sports Science Department, School of Social Sciences, Reykjavik University, IS-101 Reykjavik, Iceland; saavedra@ru.is

**Keywords:** ball throwing, hand size, arm span, motor performance

## Abstract

The objectives of this study were: (i) to analyse anthropometric parameters and throwing speed from seven meters in amateur male handball players of different ages; (ii) to know the relationship between anthropometric parameters and throwing. One hundred seventy-six male handball players (16.5 ± 5.1 years old) participated in the study, classified according to their age: senior (n = 35), U18 (n = 30), U16 (n = 37), U14 (n = 50) and U12 (n = 24). All participants were evaluated by anthropometric measurements (height, weight, body mass index, arm span, hand width) and throwing speed from 7 m standing. A one-way analysis of variance with a Bonferroni post hoc test was used to establish the differences between teams. Pearson’s simple correlation coefficients were calculated between analyse anthropometric parameters and throwing speed. Multiple linear regression was used to predict the throwing speed. Only BMI was related with throwing speed in all age groups (0.506 > r < 0.813, *p* < 0.05). Throwing speed was predicted (24–72%) with only one or two variables in each model. The selected variables were: BMI, arm span in U16 model and height U14 model, and the BMI, arm span and height are proved to be good predictors of TS in male handball players.

## 1. Introduction

Throwing the ball into the opponent’s goal is one of the most important actions for the achievement of sporting success in handball [1]. This technical-tactical gesture draws special attention as the goals difference between teams results in winners and losers at the end of a handball game [2,3,4]. It has been shown that speed and accuracy are the two most important factors while throwing the ball into the goal successfully [5]. The ball deceleration, especially when throwing further from the 9-m line, is considered a key element [6,7]. While several studies show that the increase in speed impairs accuracy [8,9].

Traditionally, it has been estimated that throwing speed (TS) depends on different factors such as technique, temporal coordination of the different body segments, and power of both upper and lower body muscle groups [10]. On the other hand, anthropometric characteristics [7,11,12], speed, and throwing accuracy [8,9] are considered as the most appropriate variables for talent detection [13].

Hand size and fingers length, influence the most on throwing in handball [14,15,16]. The latter allows a greater and better mastery of the ball [17] and seems to be the best indicator of throwing accuracy and shot due to its positive correlation with the maximum grip strength [18]. Additionally, hand span is commonly used as a reference in models identification in young handball players [19]. It has been reported that that other characteristics such as body size could also have a positive effecting handball [20]. 

Previous work have considered both throwing distances from 7 m [21,22,23] and 9 m [24,25]. This is due to their influence on the final result since it has been reported that the efficacy of the throws into the goal is one of the most important distinction between winners and losers [26]. Finally, the throwing from 7 m distances have been selected, since within the rules of handball [27] by simple easy execution and correct systematic repetition. Although there exist studies analyzing the evolution of body morphology over age in handball players, research examining the influence of those features on throwing speed at different ages is limited.

Consequently, the objectives of this study were: (i) to analyse anthropometric parameters and TS from seven meters in male handball players of different ages and, (ii) to know the relationship between anthropometric parameters and throwing speed.

## 2. Materials and Methods

A descriptive study was developed to clarify the relationship between TS (dependent variable) and anthropometric parameters (independent variables) in amateur male handball players.

### 2.1. Participants

One hundred seventy-six amateur male handball players participated in the study. Convenience sampling was by non-probability and non-random sampling. The participants were classified in function their age: senior (n = 35, 24.9 ± 5.2 yr), under-18 (n = 30, 17.13 ± 0.35 yr), under-16 (n = 37, 15.32 ± 0.47 yr), under-14 (n = 50, 13.7 ± 0.46 yr) and under-12 (n = 24, 11.25 ± 1.15 yr). All the subjects had knowledge and training experience in handball and in the technical gesture of the throw: senior (15.5 ± 5.6 yr), under-18 (8.2 ± 1, 15 yr), under-16 (7 ± 1.88 yr), under-14 (5.2 ± 1.80 yr) and under-12 (2.7 ± 1.45 yr).

All participants were informed in detail about the research protocol and the basic characteristics of the study as well as the possible risks related to the test execution, and informed consent in accordance with the Declaration of Helsinki was signed by all of them prior to the start of the study. Where needed, parents or other surrogates provided permission for under-18 and younger players. The recruitment was done among different handball teams in Aragon Handball Club, Spain, belonging to the different categories studied in the present study. The present study has the approval of the Alcala University, Spain, ethics committee.

### 2.2. Procedures

All the participants executed the same protocol under the same circumstances and were guided by a researcher. The measurements of height (m), weight (kg), and BMI (kg/m^2^) were found for every participant using a weighing scale (SECA 769; SECA Corp., Hamburg, Germany) provided with a precision stadiometer (SECA 222; SECA Corp., Hamburg, Germany). All the measurements were taken with participants wearing only underwear. The anthropometric assessment followed the guidelines issued by the International Society for the Advancement of Kinanthropometry (ISAK) [28]. All measurements were made by an ISAK Level 2 anthropometrist. A technical intraobserver measurement error of 1% was considered [29]. Arm span was measured and the distance from the edge one arm (measured at the fingertips) to the other was determined by means of a Lufkin metal anthropometric tape, standing against a flat wall, 90° arm abduction, elbows and wrists extended and palms facing forward [30]. Hand span was measured and the distance from the tip of the thumb to the tip of the little finger on the outstretched hand was determined with a metal anthropometric tape (Lufkin W606PM, Apex Tool Group, Maryland, MD, USA). All the measurements have a precision of 0.001 m.

In regards with the measurement of TS, a protocol of nine standing throws was set up using only the best result for analysis. First, a standardized warm-up established by the researchers and technical staff was performed, consisting of five min of low intensity running, three min of mobility exercises and two min of active stretching and ballistic exercises. Finally, warm-up focused on throwing was developed prior to participation. Throws were performed from the seven-meter line, allowing only one foot to be lifted and never stepping on the seven-meter line, simulating a penalty throw in handball [27], with a 30-s rest between each throw, which ensures a complete recovery [31]. The TS was recorded using a high performance sports radar (Stalker Pro 2 Radar Gun, Applied Concepts, Inc./Stalker Radar, Texas, TX, USA) placed at the 9-m line, behind the player throwing the ball, and pointing to the executing arm. Only throws that entered directly into the goal, without touching the ground, were considered as valid. Molten official handballs (Molten Corp., Hiroshima, Japan) were used, (circumference: 50–60 mm; weight: 290–475 g), depending on the regulation size corresponding to the participant’s age.

### 2.3. Statistical Analysis

The basic descriptive statistics mean and standard deviation were calculated. All the variables satisfied the tests of homoskedasticity (Levene variance homogeneity test) and normality (Kolmogorov-Smirnov test) of their distributions. One way of variance (ANOVA) was used to compare means between age groups. As significant variable effects were determined (α = 0.05), Bonferroni post-hoc pairwise comparisons were executed to determine where the main effects occurred. The intragroup linear relationships between variables pair was examined using Pearson linear correlation. A multiple linear regression was carried out to obtain the β index stepwise selection. Correlations between arm span, hand span and BMI were found via R^2^. TS was used as a dependent variable. The ranges of the variance inflation factor for all the independent variables were between 1.009 and 2.830, and they showed a small influence of collinearity. The Durbin-Watson statistic was calculated and showed that there was no autocorrelation in the residuals (the values of the statistic ranged from 1.378 to 1.627). The analysis was complemented by descriptive statistics, model fitting, estimation and confidence intervals. Relative reliability analysis was examined by the intragroup correlation coefficients (ICC). An ICC equal at or above 0.70 was considered acceptable [32]. The magnitude of between-groups differences was expressed using Cohen’s d effect size (ES) [33]. The ES adopted criteria to interpret the magnitude were as follows: trivial (<0.2), small (0.2–0.6), moderate (0.61–1.2), large (>1.2) [33]. Statistical analyses were performed using the SPSS (version 25, SPSS Inc., Chicago, IL, USA, Ill). Power analysis (post-hoc) was done using G*power software, version 3.1. Using a moderate effect size of 0.1, statistical power was 0.89 (1-beta) [34,35].

## 3. Results

**Figure 1:**
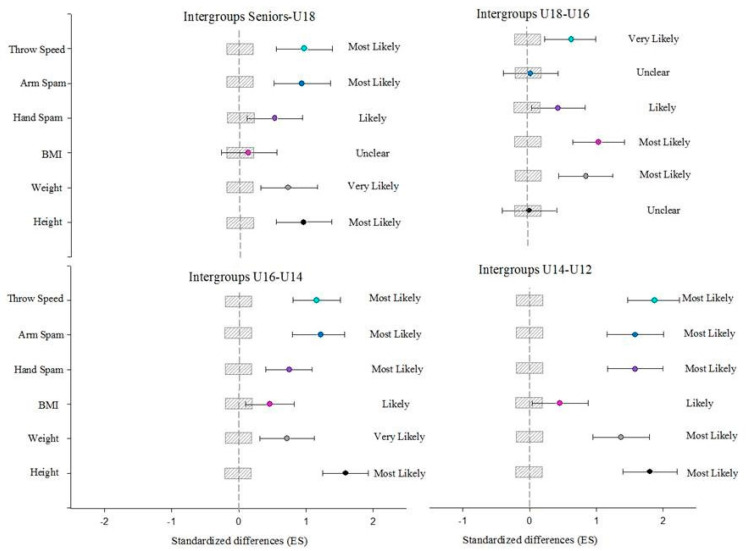
Cohen’s d effect size (ES) between-groups.

ANOVA analysis provided the differences between contiguous age groups (Table 1). Regarding TS from the 7-m line, significant differences are shown between the U16-14 (*p* < 0.001) and U14-U12 (*p* < 0.001) groups.In the intergroup analysis (Figure 1) between Senior and U18 categories, a most likely evolution is observed in the variables of TS, arm span, weight, and height. In the analysis of the relationship between U18-U16 groups, the development of BMI and weight is considered most likely. The evolution between the variables of U14 and U16 groups is considered most likely for TS, arm span, hand span and height. Ultimately, the development between U12 and U14 is considered most likely for TS, arm span, hand span, weight and height. In the intragroup analysis between Senior and U18, U16-U14 and U14-U12 categories, the relationship is considered most likely evolution. In the analysis of the relationship between U18-U16 the development is considered very likely.

A linear intragroup correlation (Table 2) was performed to clarify which variables are dependent and predictive of others, within each age group, focusing on the TS variable. In the Senior category, TS shows a significant correlation with the BMI of r = 0.514. Linear intragroup correlation for U18, taking TS as the reference variable (TS) also shows a significant correlation with BMI, with arm span, and height of r = 0.506, r = 0.478 and r = 0.431 respectively. For U16 players TS exhibits a significant correlation with BMI of r = 0.782, and with height of r = 0.55 and weight of r = 0.538. In the U14 group, the main predictor variable of TS is height of r = 0.785, BMI of r = 0.774 and weight of r = 0.576 and arm span r = 0.732 also shows a significant correlation. For U12 players, TS was also determined by the BMI of r = 0.813.

The multiple linear regression model used to predict the speed throw (Table 3) the R2 values showed that the correlation between TS and BMI in all age groups studied is confirmed except in the U16. The model predicted the TS in senior group with the BMI (β = 0.514); in U18 group with the BMI (β = 0.916); in U16 group with the arm span variable (β = 0.448); in U14 group with the BMI (β = 0.0514) and the BMI and arm span (β = 0.384); in U12 group with the BMI (β = 0.857).

## 4. Discussion

The purpose of the study was to analyse anthropometric parameters and TS from seven meters in amateur male handball players of different ages, to know the relationship between anthropometric parameters and TS.

According to the results obtained to compare age groups, can be seen how the anthropometric variables are more relevant in younger players and gradually lose their value based on their progression in the categories. Previous studies determined the anthropometric parameters at different ages [12,18,36,37]. Likewise, others analysed the relationship between throwing speed and anthropometric variables [11,23,38,39].

With respect to the differences between anthropometry and TS, differences between all age groups are shown. These data are consistent with studied the relationship between anthropometric parameters and age groups in male handball players from greater to lower involvement from the youngest to the oldest players, respectively [18], and female handball players [12,40]. There were differences between U16-U14 groups and between U14-U12 groups. The literature on this topic, in accordance with our study, shows that the game category, experience, and age contribute to the fluctuation in speed between handball players [18,41,42]. In general, the TS is mainly determined by BMI in all age groups, followed by height and arm span. For U12, the main predictive variable of TS is BMI, for U14 groups height, followed by BMI, arm span, and weight, showing the last two variables lower correlations. For U16 players, in order to establish the relation TS-anthropometry, the most influential variable is arm span. The greatest anthropometric determination of TS for U18 players is BMI, followed by arm span and height, exhibiting the last two variables a moderate relationship. In senior category, TS has a significant correlation with BMI.

Regarding the relation for each variables (Table 2) a large number of studies dealing with the relationship between TS and anthropometric [20,25,42], founding a positive effect between fat-free body mass and TS in experienced handball players and found relationship between TS and height in novice handball players. Nevertheless, the previous studies agree that the anthropometric variables are related to TS but, at the same time, the most determining anthropometric variable does not coincide in most of them. The results agree with those of [43] showed in elite players a significantly higher TS in all type of throw (standing throws and vertical jump throws), body height was significantly related to standing throws vertical jump throws only for senior athletes. Likewise, Zapartidis et al. [44] did not find any relationship with BMI, but a moderate correlation with hand length, arm span and body height was found, which was also the case in our study. In the intergroup analysis, the greatest deviation occurs in the comparison between U18-U16 groups. AT these ages, only weight and BMI show a most likely evolution in relation to TS. This might be due to the mismatches that occur at this stage of biological maturation. This leads us to think that anthropometric variables are more related to TS when we isolate the action from the competitive context. Nevertheless, other studies do not concur on the relationship between TS and BMI in handball players of similar category and level.

While interpreting the findings of the present study, some limitations need to be considered. First, the previous training experience was not considered, discarding, consequently, the likely contribution to the TS. Furthermore, the present protocol was performed by amateur handball players, thus, outcomes in elite handball players is unknown.

## 5. Conclusions

The conclusions of this study are BMI, arm span and height are proved to be good predictors of TS in amateur male handball players. However, further studies need to be undertaken in this area in order to reach a greater consensus among researchers, especially to unify what would be the anthropometric variable that mostly influences on TS in handball. The findings reported here make sport scientists and coaches clearly distinguish the different variables influencing on TS at different ages and, therefore, they facilitate their work not only on throwing improvement, but also on the return-to-play process. Hence, given that the anthropometric parameters analyzed here are easy to assess, these might be evaluated by coaches systematically over the sport career of the players allowing to know, from a practical standpoint, their evolution and the influence of body morphology on throwing speed.

## Figures and Tables

**Table 1:** Mean (M) and standard deviation (SD) of each variable. A one-way analysis of variance (ANOVA) with Bonferroni post-hot test was used to compare means between groups.

	Senior (n = 35)	U18 (n = 30)	U16 (n = 37)	U14 (n = 50)	U12 (n = 24)	F	*p*	Differences ^1^
Height (m)	1.87 ± 0.07	1.80 ± 0.06	1.80 ± 0.05	1.67 ± 0.10	1.49 ± 8.98	100.42	<0.001	U16 > U14 > U12
Weight (kg)	89.27 ± 10.45	82.06 ± 9.29	72.97 ± 11.99	58.70 ± 11.10	44.13 ± 9.52	87.80	<0.001	U16 > U14 > U12
BMI (kg/m^2^)	24.65 ± 1.63	22.97 ± 1.74	21.75 ± 1.85	19.32 ± 2.29	15.49 ± 1.67	100.69	<0.001	U16 > U14 > U12
Hand span (cm)	25.35 ± 2.55	25.04 ± 1.78	22.33 ± 3.36	20.90 ± 2.97	19.60 ± 2.94	25.45	<0.001	U18 > U16
Arm span (cm)	191.14 ± 7.71	182.41 ± 10.95	181.84 ± 7.27	170.24 ± 11.08	153.02 ± 6.52	67.05	<0.001	A > U18, U16 > U14 > U12
Throw speed (m/s)	23.78 ± 1.24	23.16 ± 1.09	22.61 ± 1.29	21.37 ± 1.96	18.74 ± 1.62	54.25	<0.001	U16 > U14 > U12

^1^ U18 = Under 18 age group; U16 = Under 16 age group; U14 = Under 14 age group; U12 = Under 12 age group; BMI = body mass index.

**Table 2:** Pearson’s linear partial correlation for each variable.

Group ^1^		Height	Weight	BMI	Hand Span	Arm Span
Senior	Height					
	Weight	0.552 **				
	BMI		0.442 *			
	Hand Span		0.750 **			
	Arm Span	0.886 **	0.563 **			
	TS			0.514 *		
U18	Height					
	Weight	0.440 *				
	BMI	0.450 *				
	Hand Span		0.902 **			
	Arm Span	0.811 **				
	TS	0.431 *		0.506 *		0.478 *
U16	Height					
	Weight	0.665 **				
	BMI	0.665 **	0.485 *			
	Hand Span		0.748 **			
	Arm Span	0.547 *		0.500 *		
	TS	0.550 *	0.538 *	0.782 *		
U14	Height					
	Weight	0.683 **				
	BMI	0.701 **	0.468 **			
	Hand Span		0.797 **			
	Arm Span	0.940 **	0.660 **	0.677 **		
	TS	0.785 **	0.576 **	0.774 **		0.732 **
U12	Height					
	Weight	0.761 **				
	BMI					
	Hand Span		0.907 **			
	Arm Span	0.916 **	0.661 *			
	TS			0.813 **		

^1^ U18 = Under 18 age group; U16 = Under 16 age group; U14 = Under 14 age group; U12 = Under 12 age group; BMI = body mass index; TS, throw speed. * *p* < 0.005; ** *p* < 0.001.

**Table 3:** Determinants of TS estimated R^2^ in different age groups in male handball players.

Age Group	R^2^	Adjusted R^2^	Constant	Determinants	Standardized β Coefficient	*p*
Senior	0.265	0.242	14.109	BMI	0.514	<0.01
Under-18	0.838	0.832	9.963	BMI	0.916	<0.01
Under-16	0.201	0.178	8.117	Arm span	0.448	<0.05
Under-14	0.679	0.666	1.315	BMI, arm span	0.514, 0.384	<0.01
Under12	0.735	0.723	3.938	BMI	0.857	<0.01

BMI = body mass index.
